# Role of Bentonite on the Mobility of Antibiotic Resistance Genes, and Microbial Community in Oxytetracycline and Cadmium Contaminated Soil

**DOI:** 10.3389/fmicb.2018.02722

**Published:** 2018-11-28

**Authors:** Honghong Guo, Shuhong Xue, Mubasher Nasir, Jialong Lv, Jei Gu

**Affiliations:** ^1^Key Laboratory of Plant Nutrition and Agri-Environment in Northwest China, College of Natural Resources and Environment, Northwest A&F University, Yangling, China; ^2^State Key Laboratory Base of Eco-Hydraulic Engineering in Arid Area, Xi’an University of Technology, Xi’an, China

**Keywords:** antibiotic resistance gene, bentonite, cadmium, human pathogenic bacteria, oxytetracycline

## Abstract

The effects of bentonite (BT), a commonly used heavy metal deactivator, on the ARGs and microbial communities in soils and lettuce systems contaminated by heavy metals and antibiotics are unclear. A study was conducted to investigate the effect of BT on the mobility of antibiotic resistance genes in oxytetracycline and cadmium contaminated soil. Results showed that the addition of BT reduced the accumulation of OTC and ARGs in the soil and lettuce roots, but increased the abundance of ARGs in lettuce leaves, and increase the risk of human pathogenic bacteria (HPB) transferring to lettuce leaves. Redundancy analysis showed that environmental factors (OTC, H_2_O, SOM, and pH) were the dominant factors that influence the distribution of ARGs and *intI1*. Network analysis showed that Proteobacteria and Bacteroidetes were the major host bacteria which caused changes in ARGs and *intI1*. There were significant positive correlations between *ermX* and *ermQ*, and a large number of HPB. The co-occurrence of *intl1* with some ARGs (*tetC, tetG, ermQ, sul1*, and *sul2*), may threaten human health due to the dispersion of ARGs via horizontal gene transfer.

## Introduction

Since their discovery, antibiotics have played important roles in the prevention and treatment of human diseases. In addition to these fundamental applications, antibiotics are used to combat animal diseases in order to ensure their growth (McManus et al., 2002; Cabello, 2006). In China, the annual amount of antibiotics used as feed additives is 8 × 10^6^ kg (Cabello, 2006). In the United States, 16 × 10^6^ kg of antibiotics are utilized each year for non-therapeutic purposes, i.e., disease prevention or improving production, where these antibiotics comprise up to 70% of the total used (Sarmah et al., 2006). These antibiotics are not fully utilized and most are excreted in animal feces and urine, and this manure is then applied to the soil, thereby causing the accumulation of antibiotics in the soil. Wang et al. (2006) reported that the oxytetracycline (OTC) content of some soils in China was as high as 200 mg/kg. Pruden et al. (2006) suggested that the long-term abuse of antibiotics leads to the multiplication of resistant bacteriain animals and their environment. In addition, a mobile genetic element “Integrons” can carry antibiotic resistance genes between non-pathogens and pathogens through horizontal gene transfer (Gyles and Boerlin, 2014; Kheiri and Akhtari, 2016). Thus, it enters the human body through food chain and endangering human health (Yang et al., 2014).

Studies have shown that the combination of pollution by antibiotics and heavy metals may impose a greater natural selection pressure to promote the spread of antibiotic-resistant bacteria (Peng et al., 2015) as well as causing the excessive expression of ARGs (Zhu et al., 2013). China is severely polluted by heavy metals, which cause an annual grain output loss of 1.0 × 10^10^ kg and a financial loss of 20 billion renminbi (Zhang et al., 2010). Ding et al. (2015) reported that cadmium was present at levels up to 6.97 mg/kg, and the proportion of ARG-positive soils with more than the standard rate of Cd was 100%. Unlike antibiotics, heavy metals do not degrade and their stress remains in the soil over the long term (Wuana and Okieimen, 2011). However, the effects of combined pollution with heavy metals and antibiotics on soil microbes and ARGs are unclear.

Montmorillonite is the major component of bentonite (BT), which is a 2:1 layered silicate. Due to interlayer van der Waals forces and charge imbalance, these layers can be penetrated easily by water and allow the cations to be balanced (Ding et al., 2009). BT is a mineral with the capacity to adsorb ions with high porosity and microbial adherence, and it is often used for environmental protection (Fernández-Nava et al., 2011). The use of BT to adsorb heavy metal ions and improve the soil environment is well known (Zou et al., 2017). We hypothesize that the high specific surface area of BT allows it to retain water and promote the passage of light, thereby increasing the hydrolysis and photolysis of OTC, and greatly reduces ARGs dispersion. However, it is unclear whether BT might have a beneficial effect on the transmission of ARGs from soil to plant under combined pollution with OTC and Cd, which may occur widely.

In this study, a commonly used vegetable “Lettuce” was grown on Cd and OTC contaminated soil. The main aims of this study were: (1) to investigate the effects of BT on the mobility and accumulation of ARGs in soil and lettuce; (2) to analyze the effects of BT on microbial community in the soil and lettuce; (3) to analyze environment factors influencing the abundances of ARGs; and (4) to identify the relationships between ARGs and bacteria (potential host bacteria of ARGs), especially human pathogenic bacteria (HPB).

## Materials and Methods

### Experimental Materials

The soil used in the experiment was collected from the top 20 cm soil layer of farmland in Yangling, China. The soil was air dried and passed through a 2 mm sieve to analyze the basic soil physio-chemical properties. Soil total nitrogen, available phosphorus and available potassium were 0.77 g/kg, 43.33, and 134.24 mg/kg, respectively. Soil pH, organic matter and cation exchange capacity were 7.27, 9.40 g/kg, and 21.42 cmol/kg, respectively, whereas soil OTC content was 0.20 mg/kg. The organic manure used in the experiment was aerobically composted pig manure collected from the experimental teaching base of Northwest A&F University, China, where the total nitrogen content was 18.50 g/kg, organic matter content was 594.65 g/kg, pH was 8.75, and OTC content was 0.81 mg/kg. However, Cd was not detected in the soil and fertilizer. OTC (purity >99%) was purchased from Solarbio (Beijing, China). The organic reagents used in the experiments were of spectral purity and the other reagents were of analytical purity. The lettuce seeds were supplied by Yangling Seed Co. The basic physico-chemical properties of soil and fertilizer were determined as described previously (Bao, 2000), including pH, organic matter (OM), total Cd, extractable Cd (bio.Cd), soil moisture content (H_2_O), total nitrogen (TN), available phosphorus (AP), available potassium (AK), and cation exchange capacity (CEC).

### Experimental Setup

Lettuce was planted in an experimental greenhouse at Northwest A&F University according to five different treatments: (1) CK = control with no added OTC and Cd; (2) O200 = 200 mg/kg OTC; (3) O200Cd = 200 mg/kg OTC + 5 mg/kg Cd; (4) BO200 = 200 mg/kg OTC + 5% BT; and (5) BO200Cd = 200 mg/kg OTC + 5 mg/kg Cd + 5% BT. We used 2% w/w pig manure to provide nutrients in order to support the lettuce growth. The amounts of OTC and Cd added were determined based on the residual concentrations found previously in soil and animal waste (Wang et al., 2006; Ding et al., 2015).

In total, 2.5 kg of soil and fertilizer were placed in plastic flowerpots with a diameter of 23 cm and a depth of 17 cm. Cd was added to the soil in the form of a CdCl_2_ aqueous solution. OTC-HCl was dissolved in distilled water and added to the soil. The prepared solution was poured into each pot, then soil was mixed thoroughly and three replicates were prepared for each treatment. The soils were allowed to equilibrate without light for 24 h. Ten lettuce seeds were sprinkled in each pot and germinated seedlings in each pot was reduced to only three plants after 10 days of germination. During the growth phase, the soil moisture content was maintained at a water-holding capacity of 60%. Lettuce plants were harvested after 55 days.

### Sample Collection

Soil and lettuce (root and leaf) samples were collected in sterile bags. The samples were stored in ice bags and transported immediately to the laboratory. The lettuce plants were rinsed with sterile water and then dried with sterile filter paper. Plant samples were divided into two parts, where one part was freeze dried to determine the OTC content and other part was frozen in liquid nitrogen to extract DNA. The soil samples were freeze–dried for DNA extraction and to determine the OTC contents.

The concentration of OTC in soil and plant sample was measured according to the previously published protocol (Boonsaner and Hawker, 2010). Briefly, 1.0 g soil or 0.5 plant sample was extracted with McIlvaine buffer–EDTA (5 ml) in an ultrasonic bath for 2 min. Sample sonication step was repeated twice, followed by filtration. Then, SPE cartridge (Strata-X) was used to pass the filtrate, and supernatant was eluted with 0.01 M oxalic acid (2 ml), and then analyzed by HPLC. The standard curve coefficient for OTC measurements was 0.98, and the recovery rate for soil and lettuce samples was 68–83%.

### DNA Extraction and High-Throughput Sequencing

Total genomic DNA was extracted from 0.5 g soil samples according to the protocol provided with a Fast DNA SPIN Kit for soil (MP Biomedicals, United States). A standard CTAB method was used to extract the plant genomic DNA with some modification (Agbagwa et al., 2012). The concentration and purity of the DNA were assessed on 1% agarose gel using a ND-2912 UV-Vis spectrophotometer (Thermo Scientific, Wilmington, DE, United States). PCR amplification was performed based on V3–V4 variable region of the bacteria 16S rRNA gene using the primer pair: 338F (ACTCCTACGGGAGGCAGCAG) and 806R (GGACTACHVGGGTWTCTAAT) (Mori et al., 2013). Sequencing was conducted by MassaBio Bio-Pharm Technology Co. (Shanghai, China) using the Illumina MiSeq PE300 platform. After removing chimeras, the high-quality sequences were clustered into operation taxonomy units (OTUs) using UPRASE at 97% similarity level. Sequences were annotated with Ribosomal Database Project (RDP) classifier^1^. The sequencing data has been uploaded to NCBI “BioProject” database having SRA accession number “PRJNA491409.” HPB were tested according to the method described by Qian et al. (2016).

### Quantitative PCR (qPCR)

ARGs often coexist in the environment at the same time and three types of ARGs (sulfonamide, tetracycline, and macrolide resistance genes) are often found in swine manure (Ji et al., 2012; Zhu et al., 2013). Therefore, we first identified the presence of the three types of ARGs using normal PCR (Bio-Rad, United States), before quantifying four tetracycline resistance genes (*tetC, tetG, tetW*, and *tetX*), two sulfonamide resistance genes (*sul1* and *sul2*), two macrolide resistance genes (*ermX* and *ermQ*), and an integrin (*intI1*) gene using the Bio-Rad IQ5 system (Bio-Rad, United States). The qPCR reaction conditions are listed in Supplementary Table S1. The absolute abundance of ARGs in each sample was calculated using the external standard curve method and 16S rRNA was quantitatively determined. The relative abundances of ARGs were determined as: ARG copy number/16S rRNA copy number.

### Statistical Analysis

Duncan’s multiple-range test (*P* < 0.05) was used to show the significance of the differences between samples using SPSS 19.0 (version 2.15.3). Network analysis was conducted using R (version 3.3.1) and Gephi software. Redundancy analysis (RDA) was performed based on ARGs and environmental factors using CANOCO (version 4.5). Other graphs were prepared using OriginPro (version 8.0) and Microsoft Excel (2016).

## Results

### OTC Concentrations in Soil and Lettuce Samples

**FIGURE 1:**
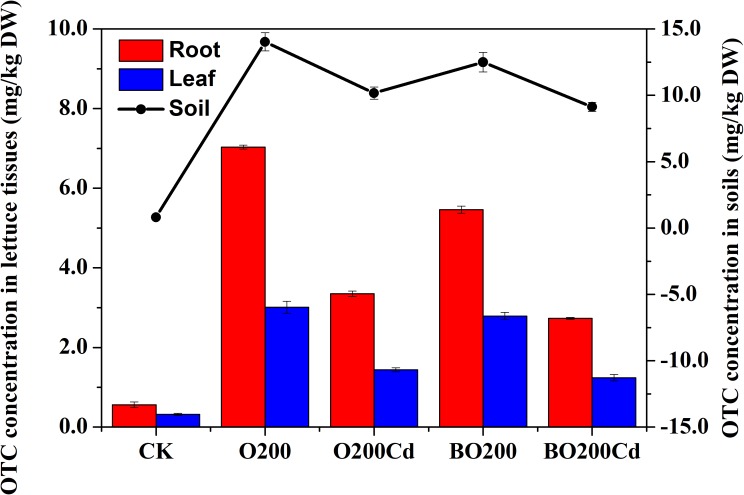
OTC concentrations in soil and leaves under different treatments.

After lettuce harvesting, we found that the residual concentration of OTC changed in the soil and lettuce samples (Figure 1). The order of OTC accumulation in the soil and lettuce samples was: soil > root > leaf. In soil, the OTC loss rate reached at 24.30–95.73% in all treatments. The application of Cd and BT significantly reduced the OTC contents in soil samples. Compared with O200, the addition of Cd significantly reduced the extractable OTC content by 27.50% in the soil (*P* < 0.05). Compared with O200 and O200Cd, the addition of BT reduced the soil OTC contents by 10.92 and 10.24%, respectively (*P* < 0.05). Similarly, the application of Cd and BT reduced the OTC contents in lettuce tissues. O200Cd reduced the OTC contents in roots and leaves by 52.35 and 51.94% (*P* < 0.05), respectively, compared with O200. When BT was added, the OTC contents of roots and leaves were decreased by 18.51–22.33 and 7.33–13.60%, respectively, compared with O200 and O200Cd (*P* < 0.05).

### Changes in the Abundances of ARGs and *intI1* in Soil and Lettuce

**FIGURE 2:**
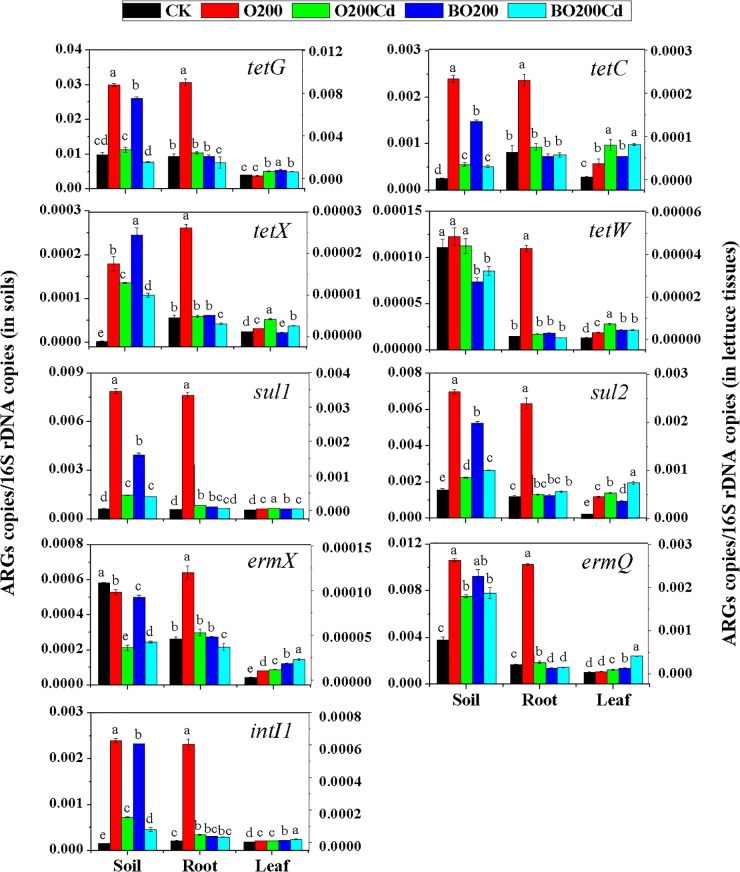
Relative abundances of ARGs in dry soil and fresh lettuce samples. Different letters within a group indicate significant differences at *P* < 0.05. Error bars indicate the SD based on three replicates.

When OTC and Cd were added, the abundance of ARGs varied significantly in the soil and lettuce tissues under each treatment (Figure 2). Overall, more ARGs were accumulated in the soil, followed by lettuce roots and leaves. The abundance of ARGs and *intI1* increased significantly in the soil and lettuce tissues with 200 mg/kg OTC (except for *tetG* and *ermQ* in leaves, and *ermX* in soil) (*P* < 0.05). Among the tetracycline resistance genes, the addition of OTC increased the relative abundance of *tetX* by 58.71, 4.78, and 0.62 times in the soil, lettuce roots and leaves, respectively. The relative abundance of *tetW* was increased by 28.03 and 2.99% in the lettuce roots and leaves, respectively, but it did not change significantly in the soil. Among the sulfonamide resistance genes, the relative abundance of *sul1* increased more than that of *sul2*, where its abundance in lettuce roots increased by 14.08 times. Among the macrolide resistant genes, the relative abundance of *ermX* did not increase in the soil, but it increased significantly in the roots and leaves.

Compared with O200, the addition of Cd reduced the abundance of all ARGs mentioned above in the soil and roots (except for *tetW* in the soil) (*P* < 0.05), but their abundance was higher than CK (except for *ermX* in soil). However, the addition of Cd significantly increased the abundance of ARGs in lettuce leaves except *intI1*, which did not show a significant difference as compared to O200 treatment.

The addition of BT significantly decreased the abundances of ARGs in soil and lettuce roots. Compared with O200, ARGs abundance was significantly lower in the soil and roots under BO200 (except for *tetX* in the soil), whereas the abundance of ARGs increased in the leaves by 0.11–2.23 times (except for *tetX* and *sul2*) (*P* < 0.05). Compared with O200Cd, BO200Cd significantly reduced the abundances of *tetG, tetX, tetW*, and *intI1* in the soil, the abundances of *tetG, tetX, sul1, ermX, ermQ*, and *intI1* in the roots, and the abundances of *tetX, tetW*, and *sul1* in the leaves (*P* < 0.05), whereas it significantly increased the abundances of *sul2, ermX, ermQ*, and *intI1* in the leaves by 0.013–3.36 times.

### Changes in the Soil Bacterial Community and Plant Endophytes

In total, 734,626 valid reads were obtained from 15 samples after filtering out the low-quality reads, removing chimeras, and pruning the linkers, adapter sequences, and primers. The average numbers of effective reads in soil, lettuce roots, and leaves were 60281, 43811, and 42832, respectively. The Venn diagram in Supplementary Figure S1 showed that 1462, 1054, and 652 OTUs were detected in the soil, lettuce roots, and lettuce leaves, respectively. The specific OTUs in soil, root and leaves were 377, 21, and 1, respectively. We characterized the abundance of bacteria based on Chao1, and the bacterial diversity using the Shannon index and Simpson index. The order of abundance and diversity of the species were: soil > root > leaf (Supplementary Table S2).

**FIGURE 3:**
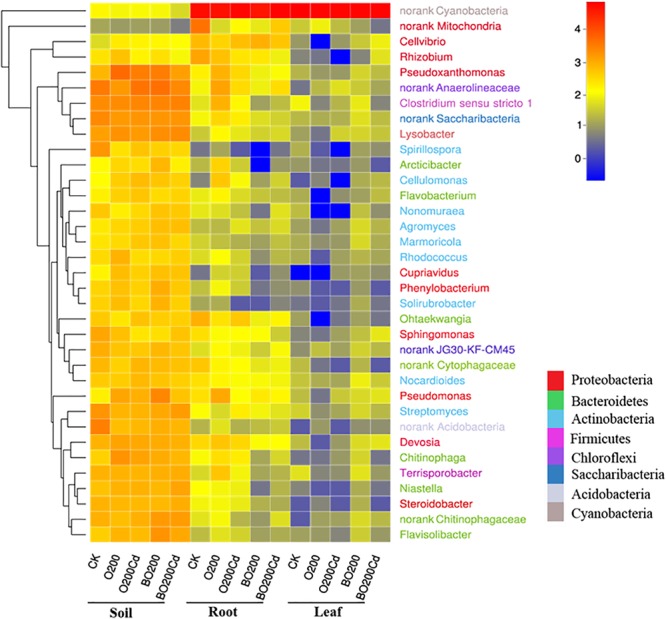
Heatmap showing the relative abundances of the top 35 genera in all samples. The 35 genera belonged to eight phyla, as shown by different colors. The shift of the bacterial community compositions are depicted by the color intensity ranged from 0 to 4.

The composition of the bacterial community changed significantly at phylum and genus levels (Figure 3 and Supplementary Figure S2). The main phyla in the samples were Proteobacteria, Bacteroidetes, Actinobacteria, Chloroflexi, and Cyanobacteria, which accounted from 70.19% of the total bacteria in the CK soil samples to 99.86% of those in the lettuce leaves under O200. Proteobacteria was the most abundant phylum in the soil and Cyanobacteria in the lettuce tissue. Compared with CK, the addition of OTC and Cd increased the abundances of Proteobacteria and Bacteroidetes in the soil by 11.30–55.68 and 47.62–68.81%, respectively, but decreased the abundances of Actinobacteria by 14.67 and 2.97%, respectively. The addition of BT reduced the abundance of Proteobacteria, Bacteroidetes, and Actinobacteria in the soil, but Chloroflexi decreased by 42.57% in O200 and increased by 4.3% in BO200 compared with CK. The changes at phylum level was caused mainly by some important genera. *Pseudoxanthomonas, Lysobacter, Pseudomonas*, and *Devosia* were the four important genera with the highest average abundances in Proteobacteria, and *Niastella, Flavisolibacter*, and *Chitinophaga* were the three genera with the highest average abundances in Bacteroidetes, where their changes in the samples were similar to those in Proteobacteria and Bacteroidetes. Compared with CK, the addition of OTC and Cd increased the abundances of these genera, but the addition of BT retarted their abundances in the soil. The microorganisms in soil, lettuce leaves, and roots exhibited different changes. Compared with CK, the addition of OTC and Cd increased the abundance of Proteobacteria in the soil, but decreased their abundance in the roots by 54.24 and 32.41% under O200 and O200Cd, respectively, and increased their abundance by 76.86 and 140.77% in the leaves under BO200 and BO200Cd.

In addition, 21 HPBs were detected in the soil and lettuce tissues (Supplementary Figure S3). *Bacteroides, Brucella, Burkholderia, Clostridium, Corynebacterium*, and *Peptoclostridium* were the main HPB detected, where they accounted for 73.69% of the total HPB. In general, more HPB were found in the soil than the lettuce plants. The addition of OTC and Cd reduced the transfer of HPB to the roots, where the abundance of HPB reduced by 16.58 and 38.16% in O200 and O200Cd, respectively. The addition of Cd increased the accumulation of HPB in leaves, and the abundance of HPB increased by 30.42% in O200Cd compared with that in O200. The addition of BT reduced the transfer of HPB by 54.35 and 44.47% to the lettuce roots, compared with the O200 and O200Cd treatments, but increased the abundance of HPB by 287.81 and 171.88% in the leaves.

### Relationships Among Environmental Factors and ARGs

**FIGURE 4:**
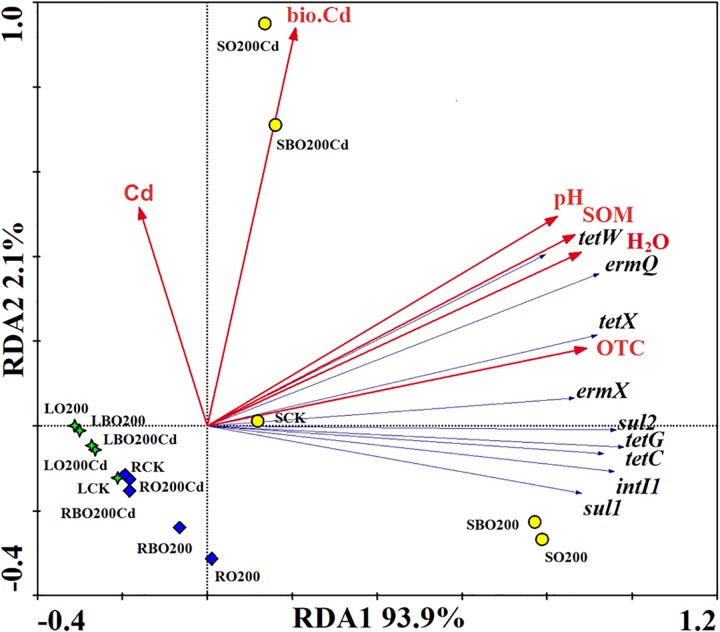
Redundancy analysis of the relationships among the main bacterial phyla, environmental factors, and ARGs (relative abundances).

The relationships among environmental factors (OTC, H_2_O, SOM, pH, bio.Cd, and Cd), and ARGs were investigated using redundancy analysis (Figure 4). Results showed that OTC had a significant effect on the distribution of ARGs, where it accounted for 25.67% of the total variance, followed by H_2_O (25.05%), SOM (24.23%), and pH (22.00%). The bio.Cd and Cd only explained 2.02 and 1.02% of the total variance. The treatments were significantly different from CK, where the application of BT made the treatment closer to CK on RD1 axis, thereby indicating that BT eliminated ARGs from the soil. The relative abundance of *tetW, ermQ, tetX*, and *ermX* explained most of the difference in the ARGs between BO200Cd and O200Cd. The relative abundance of *sul2, tetG, tetC, sul1*, and *intI1* mainly explained the differences in ARGs in soil between BO200 and O200.

**FIGURE 5:**
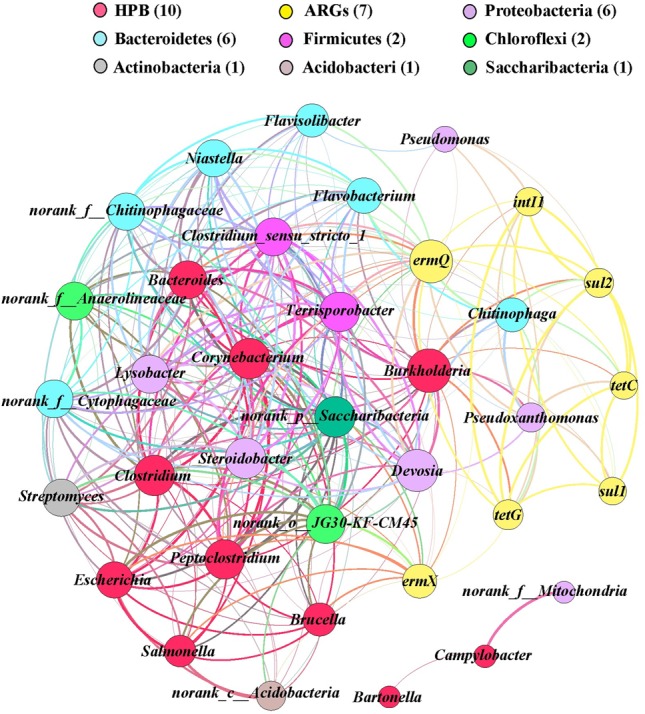
Network analysis of co-occurring ARGs (relative abundances), human pathogens bacterial, and potential host bacteria (top 30 genera) based on Pearson’s correlation coefficients (*P* < 0.01, *r* > 0.80). A node represents an ARG or bacterium, where the node size is proportional to the number of connections (degree). An edge represents a significant and strong correlation, where the edge thickness is proportional to Pearson’s correlation coefficients (weight). Different colors denote different bacterial phyla and ARGs.

In order to identify the possible host bacteria of ARGs, we employed a network analysis to determine the co-occurrence patterns of ARGs and members of the microbial communities (Figure 5). ARGs and potential host bacteria had a highly significant correlation coefficient (*r* > 0.8; *P* < 0.01). According to the network analysis, we found 25 potential host bacteria of six ARGs and *intI1*, which mainly belonged to Proteobacteria and Bacteroidetes. On average, there were 7.7 potential host bacteria per gene, where *ermX* and *ermQ* had the most host bacteria of 20 and 14, respectively. On average, each potential host bacterium contained 2.2 genes, where *Burkholderia, Pseudoxanthomonas*, and *Chitinophaga*, contained the most, with seven, six, and six genes, respectively. In addition, there were significant positive correlations between genes such as *tetG* and the other six genes. *intI1* was significantly positively correlated with *tetC, tetG, ermQ, sul1*, and *sul2*. Among the 10 HPB with higher abundance, we found 2.1 genes in each HPB. *Burkholderia* had most of the ARGs, and it had significant positive correlations with six ARGs and *intI1*. In addition, *ermX* and *ermQ* had extremely significant positive correlations with *Corynebacterium, Burkholderia, Clostridium*, and *Peptoclostridium*.

## Discussion

### Accumulation of OTC in Soil and Lettuce Tissues

After entering the environment, antibiotics are distributed in the soil, water, and air, where they are usually degraded by conversion processes such as adsorption, hydrolysis, photolysis, and microbial degradation. The degradation of antibiotics in natural ecosystems depends on the temperature, moisture, chemical composition of the environment (e.g., pH and ionic strength), and the microbial communities that contribute to biodegradation (Martinez, 2008). The half-life of OTC usually ranges from a few days to more than 1 month (Wang and Yates, 2008), with slow degradation in the winter at low temperatures (Dolliver and Gupta, 2008), and the soil composition and moisture content significantly affect its degradation (Stoob et al., 2007). In the present study, the degradation rate of OTC in soil was as high as 95.73% after 55 days. Compared with O200, the amount of extractable OTC was significantly decreased by 27.50% in O200Cd treatment, possibly due to the formation of zwitterions of OTC (Kay et al., 2010). OTC can combine with free-form heavy metal ions to form soluble complexes (Mackay and Canterbury, 2005; Wan et al., 2010), thereby reducing the extractable OTC content. However, metal ligands usually modify the functional properties of antibiotics. Cd and OTC exhibit complexation and they can modify the toxicity of antibiotics. The toxicity of the complexes formed is concentration dependent, with much greater toxicity at higher levels (Zhang et al., 2012). OTC is a tetracycline antibiotic and it is readily adsorbed by soils, clay, and sediments (Chee-Sanford et al., 2009). The adsorption mechanism mainly involves ion exchange due to the nature of tetracycline antibiotics (Figueroa et al., 2004). We found that the addition of BT significantly reduced the concentration of OTC in the soil, lettuce roots, and leaves. Due to the strong adsorption of tetracycline antibiotics, the addition of BT may change its movement in the environment (Chee-Sanford et al., 2009). BT has a high degree of water retentivity and a high specific surface area, which may promote the passage of light to increase the hydrolysis and photolysis of OTC (Genç and Dogan, 2015). In addition, the high specific surface area make the nutrients more available and provide space for microorganisms, especially antibiotic-degrading bacteria (Tang et al., 2013).

The minimum concentration of OTC in all treatments except CK was 9.13 mg/kg after 55 days, which is much higher than recommended trigger level of 100 μg/kg (VICH, 2000), although the levels in large-scale farms in China are as high as 200 mg/kg (Wang et al., 2006). These high OTC contents will increase pathogen resistance and affect human health, as well as having a negative impact on the structure and function of the soil microbial community, although this is also affected by geochemical processes involving soil elements.

### Relationships Among Environmental Factors and ARGs

Many studies have suggested that the addition of animal feces to soil system is an important route for antibiotic migration and transformation (Aga et al., 2005). The entry of such antibiotics into soil may mutate indigenous bacteria and the selective pressure imposed by antibiotics can induce the development of microbial resistance, thereby leading to the predominant growth of resistant bacteria or mutants. Gene transfer may allow drug-resistant bacteria to enter the food chain via soil–plant systems to endanger human health (Yang et al., 2014). We found that addition of OTC increased the abundance of ARGs and *intI1* in the soil and lettuce tissues, thereby suggesting that the application of organic fertilizer containing antibiotics could increase the abundances of ARGs in soil (Marti et al., 2013). Compared with O200, the addition of Cd significantly reduced the abundance of ARGs and *intI1* in the soil, except for *tetW*, but it promoted the accumulation of ARGs in lettuce leaves, mainly because the high concentrations of OTC and Cd yielded a more toxic compound that eliminated or inhibited the growth of bacteria containing these ARGs to reduce the abundance of ARGs in the soil (Flemming and Trevors, 1989). The complex formed may damage the plants, and allow the transfer of ARGs and *intI1* to the lettuce leaves. In addition, we found that BT was more effective at eliminating ARGs and *intI1* from the soil, but not from the lettuce leaves. These results suggested that other factors had a greater effect on the accumulation of ARGs and *intI1* in lettuce leaves than the concentrations of OTC and Cd, such as the changes in the bacterial community structure caused by BT (Wang et al., 2015).

Previous studies have shown that chemical properties contribute to the dynamics of the ARG through its effect on bacterial communities (Cui et al., 2018; Zhang et al., 2018). Redundancy analysis showed that OTC explained 25.67% of the variation in ARGs, thereby indicating that OTC exerted a selective pressure on the soil bacterial flora and promoted the spread of ARGs in the soil environment (Peng et al., 2015; Sandberg and Lapara, 2016). Heavy metals can kill or inhibit the bacterial hosts of ARGs, thereby reducing the abundances of ARGs (Qian et al., 2016). BT affects microbial communities either by decreasing the bioavailability of Cd, or by influencing the soil pH and available nutrient levels (Dias et al., 2010; Wang et al., 2012), which also change the abundance of ARGs.

Using network analysis, we found that the potential host bacteria of ARGs mainly belonged to the phyla Proteobacteria and Bacteroidetes, where the four genera with the highest average abundances in Proteobacteria (*Pseudoxanthomonas, Lysobacter, Pseudomonas*, and *Devosia*) and the three genera with the highest average abundances in Bacteroidetes (*Niastella, Flavisolibacter*, and *Chitinophaga*) had significant positive correlations with multiple ARGs, thereby indicating that the changes in the bacterial community mainly explained the changes in ARGs, where multiple ARGs could have the same host bacteria (Li et al., 2015). There were also correlations between the potential host bacteria, and thus the presence of these genes may be driven by a common resistance mechanism (Baker-Austin et al., 2006).

Integrase genes are important for gene transfer factors and they are generally considered to be indicators of horizontal gene transfer (Fluit and Schmitz, 1999; Chen et al., 2014). We found that the addition of OTC and Cd significantly increased the relative abundance of *intI1* in the soil and lettuce tissues, which suggests that it might lead to the generation of multi-drug resistance genes. The presence of these multi-drug resistance genes in the human body may lead to the generation of resistant bacteria and threaten human health. There were also significant positive correlations between *intI1* and *tetC, tetG, ermQ, sul1*, and *sul2*, thereby suggesting that *intI1* may be involved with the horizontal transfer of resistance genes between microorganisms to cause multiple resistance. The significant correlations between ARGs may be due to their localization in the same host bacteria or genetic elements, such as plasmids and insertion elements (Agersø and Sandvang, 2005; Toleman et al., 2006).

ARGs are derived from environmental bacteria (Davies, 1994; Alonso et al., 2001). However, resistance genes are often present in HPB when there is no high antibiotic loading (Pallecchi et al., 2008). As these genes enter the genetic transfer transposons, their transmission speed is greatly increased in natural ecosystems, and they have potentially more detrimental effects on humans and environmental systems than the antibiotics themselves. We found that *ermX* and *ermQ* had the most potential host bacteria as well as significant positive correlations with some HPBs such as *Corynebacterium, Burkholderia, Clostridium*, and *Peptoclostridium*. It has been reported that *erm* genes are transmitted by plasmids and transposons in Gram-positive and Gram-negative bacteria (Roberts, 2008). Kresken et al. (2004) showed that the *erm* gene family is present in *enterococci, streptococci, Streptococcus pneumoniae*, and *Campylobacter jejuni* with different origins. It should be noted that the addition of BT reduced the abundance of HPB in lettuce roots but increased their abundance in lettuce leaves, which indicates that the application of BT to heavy metals and antibiotics contaminated soil need further exploration in the field condition.

## Conclusion

In this study, addition of OTC mostly increased the abundance of ARGs and *intI1* in the soil and lettuce tissues. The addition of Cd and BT reduced the accumulation of OTC and ARGs in the soil and lettuce roots, but the abundances of ARGs increased in the lettuce leaves and the transfer of HPB to lettuce tissues was enhanced. Redundancy analysis showed that environmental factors greatly influence the changes in ARGs and *intI1*. The changes in Proteobacteria and Bacteroidetes strongly influenced the changes in ARGs and *intI1*. There were significant positive correlations between *ermX, ermQ*, and a large number of HPB, including *Corynebacterium, Burkholderia, Clostridium*, and *Peptoclostridium*. The higher abundance of *intI1* in soils and lettuce, and its co-occurrence with some ARGs may represent a threat to human health due to dispersion of ARGs via horizontal gene transfer.

## Author Contributions

HG and JL designed the experiments. HG and SX conducted the experiments. HG and MN wrote the manuscript. MN and JG revised the manuscript.

## Conflict of Interest Statement

The authors declare that the research was conducted in the absence of any commercial or financial relationships that could be construed as a potential conflict of interest.

## Footnotes

1 http://rdp.cme.msu.edu/
