# Supplementary material for: Role of Bentonite on the Mobility of Antibiotic Resistance Genes, and Microbial Community in Oxytetracycline and Cadmium Contaminated Soil
Source: Front Microbiol. 2018 Nov 28;9:2722. doi: 10.3389/fmicb.2018.02722 (PMC6279858; doi:10.3389/fmicb.2018.02722)
Supplement: Supplementary file 1 [file Table_1.pdf]

Supplementary materials for

Role of bentonite on the mobility of antibiotic resistance genes, and microbial community in oxytetracycline and cadmium contaminated soil

Authors: Honghong Guo[1], Shuhong Xue[2], Mubasher Nasir[1], Jialong Lv[1]*, Jei Gu

***Corresponding author:** Jialong Lv

Email: ljlll@nwsuaf.edu.cn

Address:

1. College of Natural Resources and Environment, Key Laboratory of Plant Nutrition and Agri-environment in Northwest China, Northwest A&F University, Yangling, Shaanxi 712100, China;

2. State Key Laboratory Base of Eco-Hydraulic Engineering in Arid Area, Xi'an University of Technology, Xian 710048, China

The qPCR reaction mixture comprised 1 μL of DNA template, 0.25 μL of each 20 pM primer (ShengGong, China), 10 μL of SuperReal PreMix Plus (TianGen, China), and 8.5 μL of RNase-free water. The qPCR conditions comprised an initial hold for 15 min at 95 °C, followed by 40 cycles for 10 s at 95 °C, 20 s at the annealing temperature, and then 32 s at 72 °C. To eliminate the effects of inhibitory compounds, the DNA template was a tenfold dilution of extracted DNA. qPCR was performed using Bio-Rad IQ5 (Bio-Rad, USA).

Table S1. PCR primers used in this study

Gene name	Primer	Size (b)	Annealing temperature (°C)	References
tetC	F: GCGGGATATCGTCCATTCCG<br>R: GCGTAGAGGATCCACAGGACG	207	59	(Aminov et al., 2002)
tetG	F: GCAGAGCAGGTCGCTGG<br>R: CCYGCAAGAGAAGCCAGAAG	134	54	(Aminov et al., 2001)
tetW	F: GAGAGCCTGCTATATGCCAGC<br>R: GGGCGTATCCACAATGTTAAC	168	56	(Aminov et al., 2001)
tetX	F: CAATAATTGGTGGTGGACCC<br>R: TTCTTACCTTGGACATCCCG	468	55	(Ng et al., 2001)
sul1	F: CGGCGTGGGCTACCTGAACG<br>R: GCCGATCGCGTGAAGTTCCG	433	60	(Frank et al., 2007)
sul2	F: GCGCTCAAGGCAGATGGCATT<br>R: GCGTTTGATACCGGCACCCGT	293	59	(Frank et al., 2007)
ermX	F: GAGATCGGRCCAGGAAGC<br>R: GTGTGCACCATCGCCTGA	488	61	(Chen et al., 2007)
ermQ	F: CACCAACTGATATGTGGCTAG<br>R: CTAGGCATGGGATGGAAGTC	154	60	(Koike et al., 2007)
intI1	F: CTGGATTTCGATCACGGCACG<br>R: ACATGCGTGTAAATCATCGTCG	473	60	(Frank et al., 2007)
16S V3	F: CCTACGGGAGGCAGCAG<br>R: ATTACCGCGGCTGCTGG	193	55	(Aminov et al., 2002)

Table S2. Alpha diversity index of microbial community

Treatment		Shannon	Simpson	Chao1
Soil	CK	6.103958	0.00543	1375.1891
	O200	5.745832	0.01071	1325.8636
	O200Cd	5.776404	0.008696	1297.4315
	BO200	5.752587	0.009072	1328.6
	BO200Cd	5.827118	0.007705	1332.2262
Root	CK	1.580833	0.58799	827.375
	O200	1.976253	0.548823	1066.7431
	O200Cd	1.055837	0.76041	753
	BO200	0.822723	0.805363	624.38095
	BO200Cdd	0.639988	0.857318	682.8875
Leaf	CK	0.157001	0.968731	459.35714
	O200	0.081194	0.984336	413.53571
	O200Cd	0.11372	0.978306	494.23809
	BO200	0.295225	0.942251	716.46753
	BO200Cd	0.253339	0.947041	439

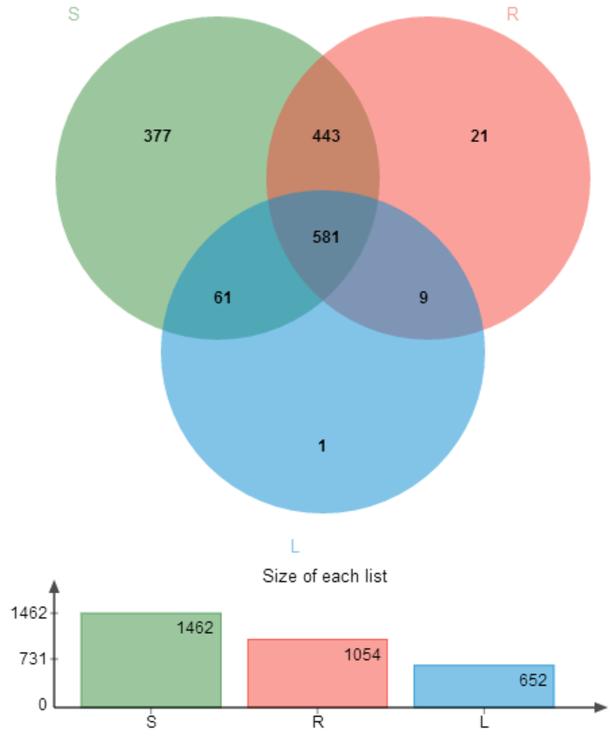

Fig. S1. Venn diagram showing the amounts of bacterial OTUs in soil (S), lettuce roots (R), and leaves (L).

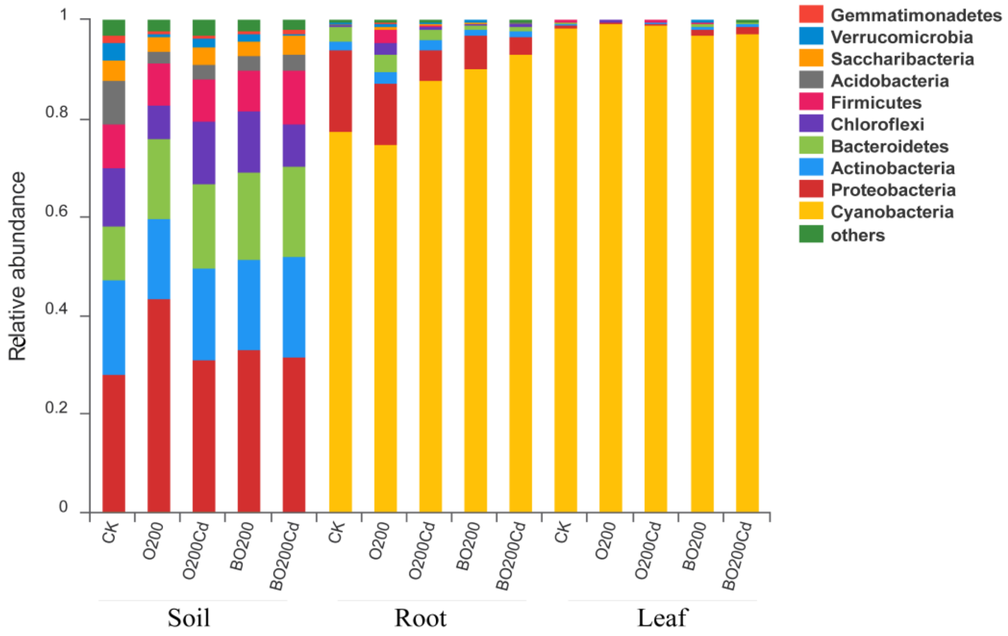

Fig. S2. Taxonomic classification of bacterial reads retrieved from different samples at phylum level using RDP classifier. Others represent the relative abundance of all other phyla outside the 10 phyla.

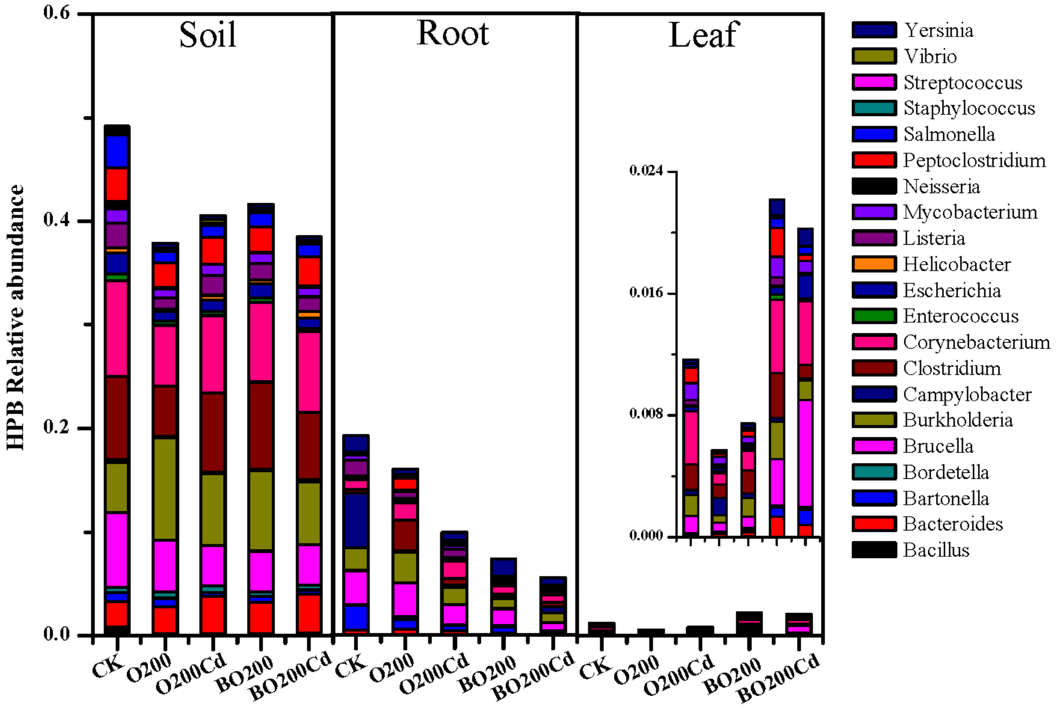

Fig. S3. Relative abundances of 21 human pathogenic bacteria in soil and lettuce samples.

References:

Aminov, R. I., et al., 2002. Development, Validation, and Application of PCR Primers for Detection of Tetracycline Efflux Genes of Gram-Negative Bacteria. Appl Environl Microb, 68: 1786-1793.

Aminov, R.N., et al., 2001. Molecular Ecology of Tetracycline Resistance: Development and Validation of Primers for Detection of Tetracycline Resistance Genes Encoding Ribosomal Protection Proteins. Appl Environ Microb, 67: 22-32.

Chen, J., et al., 2007. Development and application of real-time PCR assays for quantification of erm genes conferring resistance to macrolides-lincosamides-streptogramin b in livestock manure and manure management systems. Appl Environ Microb, 73: 4407-4416.

Frank, T., et al., 2007. Characterization of sulphonamide resistance genes and class 1 integron gene cassettes in Enterobacteriaceae, Central African Republic (CAR). J Antimicrob Chemotherapy, 59: 742-745.

Ng, L. K., et al., 2001. Multiplex PCR for the detection of tetracycline resistant genes. Mol Cell Probe, 15: 209-215.

Koike, S., et al., 2010. Molecular ecology of macrolide – lincosamide– streptogramin B methylases in waste lagoons and subsurface waters associated with swine production. Microb Ecol, 59(3): 487-498.
